# Endothelial Protein C Receptor gene Expression in a Female with Homozygous EPCR gene 23-bp Insertion

**DOI:** 10.5152/tjh.2011.47

**Published:** 2012-03-05

**Authors:** Afife Karabıyık, Nejat Akar

**Affiliations:** 1 Ankara University, School of Medicine, Department of Pediatric Molecular Genetics, Ankara, Turkey

## TO THE EDITOR

Endothelial protein C receptor (EPCR) is an essentialcomponent of the protein C (PC) anticoagulant pathwayand is important for regulation of coagulation [1,2,3]. EPCRhas a transmembrane domain, extracellular domains, anda very short cytoplasmic tail, and is primarily localized onthe endothelial cells of large blood vessels. The humanEPCR gene is located on chromosome 20q11.2, and has 4exons and 3 introns [4,5]. EPCR has both an endothelialcell-specific transmembrane form and a soluble form thatarises via metalloprotease cleavage [6].

To date, several polymorphisms and mutations—including a 23-bp insertion—have been reported on thehuman EPCR gene. The EPCR gene 23-bp insertion TATCCACAGTTCCTCTGACCATCis located between intron2 and exon 3 (nt4031), and is related to thrombotic riskand myocardial infarction [7,8,9,10]. Exons 2 and 3 encodemost of the extracellular region of the EPCR gene [5]. Thisinsertion of 23 nucleotides preceding the insertion point(nt4031) introduces a frameshift and premature stop thatdeletes the entire alpha 2 domain of the gene [10]. Thetruncated protein results in absence of the cytoplasmictail, transmembrane domain, and part of the extracellulardomain. As such, this mutation is probably a good modelfor an EPCR null-allele.

Homozygous null mice embryos died prior to embryonicd 10.5, and it was reported that EPCR is essential fornormal embryonic development and plays a key role inpreventing thrombosis at the maternal-embryonic interface[11,12]. The homozygous state of EPCR gene 23-bpinsertion is very rare, and we only reported it once beforein a 8-month-old boy with sepsis [13].

Herein we report a 25-year-old female with homozygous23-bp insertion of the EPCR gene. The patient hadexperienced abortus twice, and then gave birth followingantiplatelet (aspirin) therapy. She was referred for evaluationof thrombotic risk factors to us. The family historywas negative for thrombotic disease. Informed consentwas provided by the patient. Her DNA was examined forfactor V 1691 and prothrombin 20210 mutations, andshe carried normal alleles. Factor VIII, factor IX, proteinC, antithrombin, protein S, homocysteine, and lipoprotein(a) levels were normal. Plasma sEPCR was 227 ngmL^–1^ (38-132 ng mL^–1^), which was measured via enzymelinkedimmunosorbent assay (ELISA) (Diagnostica StagoAsserachrom sEPCR, Asnieres-France). EPCR gene exon 3amplification was performed using primers 5’-ACACCTGGCACCCTCTCT-3’ and 5’CATCCTTCAGG TCCATCC-3’at an annealing temperature of 58 °C. To detect the 23-bpinsertion the PCR product was electrophoresed in 3% agarosegel and stained with ethidium bromide. The patient was homozygous for the EPCR gene 23-bp insertionmutation, and her father, mother, and child were heterozygousfor the insertion.

RNA was isolated from blood samples obtained fromthe index case and a control, and then the level of expressionof EPCR mRN A was determined (Roche Light Cycler1.5, Basel, Switzerland), following RN A isolation andcDNA synthesis (Roche, Switzerland). Quantitative realtime(RT)-PCR was used to measure gene expression usingEPCR-specific fluorescent marked UPL probes (Probe50) and primers (EPCRF 5’-GTAGCCAAGACGCCT-3’,EPCRR 5’-GATAGGGG TCGCGG A-3’) (Roche, Switzerland).The glyceraldehyde-3-phosphate dehydrogenase(GAPDH) housekeeping gene was used for normalizationof EPCR gene expression data. All experiments were performedtwice. Statistical analysis of the results was performedusing two-way ANOVA (GraphPad Prism v.5.00,GraphPad Software, San Diego, California, USA, http://www.graphpad.com)

**Figure 1:**
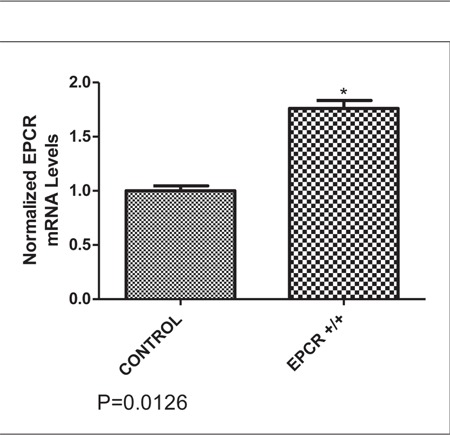
The EPCR mRN A level in the patient (1.7-foldhigher) and control.

The patient’s EPCR mRN A level was 1.7-fold higherthan that of the control, as shown in the Figure 1. Becauseof the premature stop of the EPCR protein, which was dueto the 23-bp homozygous insertion, her EPCR expressionlevel could be higher than the control that has normalEPCR gene 23-bp mutation allele. This can be explainedby the EPCR protein requirement.

Disruption of the EPCR gene in mice leads to earlyembryonic death [12]. The presented case is not only alive, but also gave birth to a healthy child. As the patienthad abortus twice, we think that homozygous 23-bpinsertion might affect the fetus negatively by causinghypercoagulability. There is a strong association betweenanti-EPCR autoantibodies and the risk of fetal death. Highlevels of IgM and IgG anti-EPCR in humans are associatedwith a high risk of a first episode of fetal death [14].EPCR gene 23-bp insertion in women with fetal loss ismore prevalent than in women that have given birth to ≥1healthy baby and have no history of late fetal death [11].The data obtained in the presented case show that 23-bphomozygous mutation of the EPCR gene in humans iscompatible with life. Additional research—including caseswith homozygous 23-bp insertion mutations is neededto clarify the possible effects of the insertion.

## CONFLICT OF INTEREST STATEMENT

The authors of this paper have no conflicts of interest,including specific financial interests, relationships, and/or affiliations relevant to the subject matter or materialsincluded.
